# Lingual Gyrus Surface Area Is Associated with Anxiety-Depression Severity in Young Adults: A Genetic Clustering Approach

**DOI:** 10.1523/ENEURO.0153-17.2017

**Published:** 2018-01-19

**Authors:** Baptiste Couvy-Duchesne, Lachlan T. Strike, Greig I. de Zubicaray, Katie L. McMahon, Paul M. Thompson, Ian B. Hickie, Nicholas G. Martin, Margaret J. Wright

**Affiliations:** 1 Queensland Brain Institute, University of Queensland, Brisbane 4072, Australia; 2 QIMR Berghofer Medical Research Institute, Brisbane 4006, Australia; 3 Centre for Advanced Imaging, University of Queensland, Brisbane 4072, Australia; 4 Institute of Health Biomedical Innovations, Queensland Institute of Technology, Brisbane 4006, Australia; 5 Imaging Genetics Center, Keck School of Medicine, University of Southern California, Marina del Rey, CA 90292; 6 Brain and Mind Research Institute, University of Sydney, Sydney 2050, Australia

**Keywords:** cortical surface area, depression anxiety, endophenotype, genetic clustering, lingual gyrus, nonlinear effect

## Abstract

Here we aimed to identify cortical endophenotypes for anxiety-depression. Our data-driven approach used vertex-wise genetic correlations (estimated from a twin sample: 157 monozygotic and 194 dizygotic twin pairs) to parcellate cortical thickness (CT) and surface area (SA) into genetically homogeneous regions (Chen et al., 2013). In an overlapping twin and sibling sample (*n* = 834; aged 15–29, 66% female), in those with anxiety-depression Somatic and Psychological Health Report (SPHERE) scores (Hickie et al., 2001) above median, we found a reduction of SA in an occipito-temporal cluster, which comprised part of the right lingual, fusiform and parahippocampal gyrii. A similar reduction was observed in the Human Connectome Project (HCP) sample (*n* = 890, age 22–37, 56.5% female) in those with Adult Self Report (ASR) DSM-oriented scores (Achenbach et al., 2005) in the 25–95% quantiles. A *post hoc* vertex-wise analysis identified the right lingual and, to a lesser extent the fusiform gyrus. Overall, the surface reduction explained by the anxiety-depression scores was modest (*r* = −0.10, 3rd order spline, and *r* = −0.040, 1st order spline in the HCP). The discordant results in the top 5% of the anxiety-depression scores may be explained by differences in recruitment between the studies. However, we could not conclude whether this cortical region was an endophenotype for anxiety-depression as the genetic correlations did not reach significance, which we attribute to the modest effect size (*post hoc* statistical power <10%).

## Significance Statement

Endophenotypes may help shed light on the etiology, cognitive impairment and genetics of psychiatric disorders. Here, we report a nonlinear negative association between anxiety-depression and smaller surface area (SA) of the occipito-temporal region, which comprises most of the right lingual and fusiform gyri (*n* = 834). This cluster was defined by applying a fuzzy clustering algorithm to a matrix of vertex-wise genetic correlations among cortical surface measures. We replicated this association in an independent sample from the Human Connectome Project (HCP; *n* = 890). We could not confirm the presence of a genetic correlation as the effect size of the association was modest (*r* = −0.10).

## Introduction

Endophenotypes may be useful in decomposing or deconstructing a psychiatric disorder, which should result in more successful genetic analyses, redefinition of diagnosis, improved studies of the course of illness and development of pertinent animal models (Gottesman and Gould, 2003). According to a widely accepted definition, an endophenotype needs to satisfy four core criteria (Gottesman and Gould, 2003): it should be heritable, genetically and phenotypically associated with the illness in the population and state independent (not a consequence of the disorder). There should also be a genetic correlation between the disease and endophenotype: unaffected family members of affected individuals should also exhibit the endophenotype, to some extent, as they have a higher genetic load for the disease than people who are not closely related to affected individuals. Reliability of the endophenotype is sometimes included as part of the definition (Goldstein and Klein, 2014) and is implied by the trait heritability (Dohm, 2002).

The endophenotype concept is popular (Glahn et al., 2014), but only a few endophenotypes have been identified for affective disorders (Glahn et al., 2012; Goldstein and Klein, 2014; Bas-Hoogendam et al., 2016). These include: neuroticism (Goldstein and Klein, 2014), personal life events (Boardman et al., 2011) and perceived social support (Kendler and Baker, 2007; Matthews et al., 2016). Magnetic resonance imaging (MRI) brain measures may be promising endophenotypes, as they are objective (not self-reported), reliable (Liem et al., 2015), and heritable (Strike et al., 2015; Whelan et al., 2016). There has been an explosion of proposed brain endophenotypes for depression, and most publications report unreplicated phenotypic associations based on small sample sizes (for review, see Savitz and Drevets, 2009; Hasler and Northoff, 2011; Zhang et al., 2013). To overcome publication bias and propose robust candidate endophenotypes, the major depressive disorder (MDD) group of the ENIGMA consortium (Thompson et al., 2014) has conducted large-scale case-control comparisons, which found several structural brain phenotypes consistently associated with MDD (Schmaal et al., 2016, 2017b), but somewhat conflicting cortical signatures of depression when stratifying the analysis by age (cutoff 21 years). In addition, another large voxel-wise meta-analysis of cortical gray matter density reported a profile of volume changes associated with MDD (Arnone et al., 2016). However, more work is needed to determine genetic relationships between depression and structural brain measures and to replicate the reported associations.

Here, we aimed to identify cortical thickness (CT) or surface area (SA) endophenotypes for depression and anxiety, which share most of their genetic risk (Kendler et al., 1987; Hettema, 2008). We considered both CT and SA, as there is evidence they are influenced by specific environmental and genetic factors, and thus are preferred to combined volumetric measures as they gather more information (Panizzon et al., 2009; Winkler et al., 2010). We performed an exploratory data driven analysis in a large, genetically informative young adult twin sample, followed by a replication in an independent cohort of similar age. The large cohorts allowed us to investigate the presence of nonlinear relationships between cortical structures and anxiety-depression level.

We used twin modeling (Martin and Eaves, 1977; Neale and Cardon, 1992) to perform a genetic parcellation of the cortex (Chen et al., 2013), aiming to reduce the data dimension, while ensuring genetic homogeneity of the brain phenotypes studied. We controlled for mean SA/CT to adjust for the confounding effect of overall brain or body size. To measure anxiety-depression, we used a validated continuous measure of anxiety-depression [Somatic and Psychological Health Report (SPHERE); Hickie et al., 2001] collected before the MRI scan, rather than a DSM-IV lifetime diagnosis (with disease onset not always preceding imaging). We could then get around the question of state-independence (even if a longitudinal design is needed to confirm the direction of causation) and maximized statistical power by providing a larger sample with greater variance across the population. In the replication sample, we used validated Achenbach scales (Achenbach et al., 2005; Achenbach, 2009) collected at the time of imaging. Furthermore, we aimed to characterize sources of covariation between anxiety-depression and cortical measures using twin modeling.

## Materials and Methods

### Exploratory analysis

#### Participants

We analyzed data from a total of 834 twins and siblings from the Brisbane Longitudinal Twin Study (BLTS; Wright and Martin, 2004; Gillespie et al., 2013) who were assessed for symptoms of anxiety-depression and who had undergone brain imaging. Anxiety-depression was assessed at mean age 17 (SD = 2.2, range 10–24) with imaging completed 4.4 years (SD = 2.1, range 0.6–9.5 years) later, at a mean age of 21 (SD = 3.2, range 15–29). The sample (66% female) comprised 23 twin-sibling trios (9 MZ, 14 DZ), 275 complete pairs (101 MZ, 142 DZ and 32 pairs of siblings treated as DZ) and 214 singletons (Table 1). Zygosity of twin pairs was initially determined from DNA using a commercial kit (AmpFISTR Profiler Plus Amplification kit, ABI) and was later confirmed by genome-wide single nucleotide polymorphism genotyping (Illumina 610K chip). Informed consent was obtained from all participants, including a parent or guardian for those aged <18 years. Participants received an honorarium for each study. The studies were approved by the Human Research Ethics Committees of the QIMR Berghofer Medical Research Institute, the University of Queensland and Uniting Health Care.

**Table 1.** Detailed family structure of the QTIM and HCP samples

			**QTIM**	**HCP**
Total sample size (individuals) for phenotypic analysis			834	890
Final sample size (individuals) for twin modeling			833^*^	853**‡
Incl.	*N* complete trios		23	184
	Incl.	*N* MZ pairs + one sibling	9	75
		*N* DZ pairs + one sibling	14	71
		*N* sibling trios	0	38
	*N* complete pairs		275	109
	Incl.	*N* MZ pairs	101	14
		*N* DZ pairs	142	19
		*N* twin-sib pairs	32	38
		*N* sibling pairs	0	38
	*N* singletons		214	83
	Incl.	*N* twins	181	33
		*N* non-twin siblings	33	50

* One individual from a family of four siblings was excluded for twin modeling, i.e., maximum family size restricted to three individuals (twin and/or non-twin siblings).

** Thirty-seven individuals from families with more than four siblings were excluded with no effect on the sample characteristics (mean age 28, SD = 3.7, range 22–37, 56.5% females after exclusion).

‡ To maximize the HCP sample, 12 half-siblings were included (categorized as siblings in twin analysis); this low number should not affect estimates from twin modeling.

#### Brain imaging

Imaging was conducted using a 4T Bruker Medspec whole-body MRI on 1161 twins and their siblings aged 15–30 (62% female), as part of the QTIM study of brain structures and function (de Zubicaray et al., 2008; Blokland, 2014; Rentería et al., 2014). Participants were all right-handed and were screened for prior mental health diagnoses and anti-depressant use, as well as neurologic disorders and loss of consciousness. Structural T1-weighted 3D images were acquired with the following parameters: TR = 1500 ms, TE = 3.35 ms, TI = 700 ms, 240 mm FOV, 0.9 mm slice thickness, 256 or 240 slices depending on acquisition orientation: 86% coronal (256 slices), and 14% sagittal (240 slices). The raw T1-weighted images were corrected for intensity inhomogeneity with SPM8 (Flandin and Friston, 2008; Wellcome Department of Imaging Neuroscience, 2009). Cortical surfaces were reconstructed using FreeSurfer (v5.3; Fischl and Dale, 2000; Fischl, 2012), then resampled into a common space, smoothed (*n* = 2819 iterations, nearest neighbor) and down-sampled, with SA and CT measured at each surface vertex (2562 per hemisphere). Whole-brain total SA and mean CT were additionally extracted.: From the total imaging sample (*n* = 1161), we excluded 11% of participants due to neuroanatomical abnormalities (*n* = 48), excessive head motion during scanning (*n* = 16), or poor quality FreeSurfer cortical surface reconstructions (*n* = 57, using the ENIGMA quality checking procedure that consists of manual QC assisted by automatic detection of outliers; enigma.ini.usc.edu).

#### Assessment of anxiety-depression

The anxiety-depression score was calculated using the SPHERE questionnaire (Hickie et al., 2001), which has been administered to 3312 twins and siblings across one to four waves of the BLTS. When several SPHERE scores were available, we selected the closest to the time of scan. We previously showed that this score is moderately heritable (Hansell et al., 2012) and has good psychometric properties (stochastic ordering on the sum score, three months’ test-retest, internal consistency, limited sex bias; Couvy-Duchesne et al., 2017). In addition, we have shown that the anxiety-depression score from the SPHERE collected in adolescence predicts lifetime DSM-IV MDD diagnoses in young adulthood in the BLTS sample.

#### Genetic parcellation of the cortex

Twin modeling contrasts MZ twins (who share the same genetic information and familial environment) with DZ twins (who have the same familial environment but share on average half of their genetic information) and unrelated pairs (independent familial environment and genetic information). It allows the estimation of the proportion of interindividual differences attributable to genetic variability in the population [narrow sense heritability or additive genetic effects (A), the individuals’ unique environment (E), and either the familial environment (C) or genetic dominance (D; Martin and Eaves, 1977; Neale and Cardon, 1992; Verweij et al., 2012]. Multivariate models can further break down the sources of variances into common and trait specific, providing an estimate of genetic and environmental correlations (Martin and Eaves, 1977; Neale and Cardon, 1992).

Following prior work (Chen et al., 2013), and to reduce the number of brain phenotypes tested (and the burden of multiple testing correction), we estimated the matrices of genetic correlations between the vertex-wise SA and CT measures (2305 left hemisphere, 2308 right hemisphere; medial wall vertices excluded) within each hemisphere. To do this, we used bivariate AE models (Neale and Cardon, 1992) using OpenMx (Boker et al., 2011), which implements Full Information Maximum Likelihood (FIML) and allows some missingness in the outcome variables. Omitting C/D terms in the models may result in a slight overestimation of the genetic correlation, as only moderate shared environmental sources of variance have been detected throughout the cortex (Lenroot et al., 2009; Kremen et al., 2010; Eyler et al., 2011). We included a maximum of three individuals (e.g., one twin pair and a sibling) per family. This increases power to detect A and C/D compared to the standard twin design (Posthuma and Boomsma, 2000).

**Figure 1.**
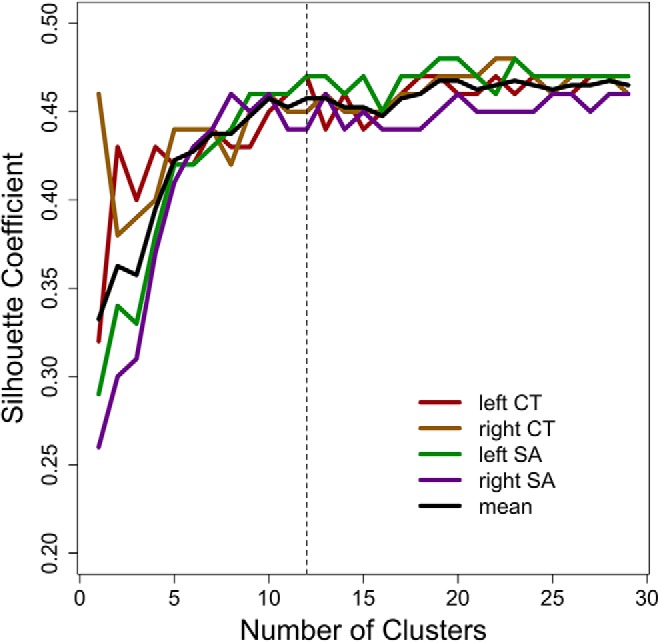
Silhouette coefficients of the clustering scenarios. Vertical dashed line corresponds to 12 clusters per hemisphere and measurement, which we used to parcellate the cortex.

**Figure 2.**
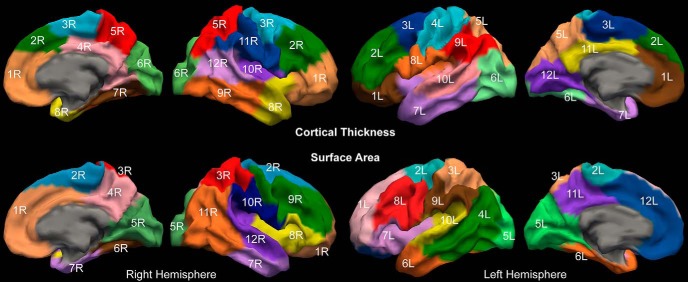
Summary of genetic parcellation of the CT and SA. Clusters are labeled 1L to 12L for the left hemisphere and 1R to 12R for the right one. Extended Data Figures 2-1, 2-2, 2-3, 2-4 describe the genetic clusters used in this analysis.

Before estimating the genetic correlations, we residualised the CT and SA measures to remove global effects (whole-brain total SA, mean CT), as well as effects of sex, age, acquisition orientation. We used a fuzzy clustering algorithm to identify clusters of genetically correlated voxels (Kaufman and Rousseeuw, 1990; Chen et al., 2013) implemented in the R package “cluster” (R Development Core Team, 2012; Maechler et al., 2016) and determined the optimal number of clusters using silhouette coefficients (Kaufman and Rousseeuw, 1990; Chen et al., 2013; Fig. 1). Such coefficients combine cluster cohesion (intra cluster differences) and separation (inter cluster differences). A high coefficient indicates better-separated clusters (Chen et al., 2013). The silhouette coefficients plateaued after 10–14 clusters, for each hemisphere and measurement (Fig. 1). We restricted our analyses to 12 clusters, which results in a relatively parsimonious parcellation, to facilitate both comparisons across hemisphere and with prior work (Chen et al., 2013). For each cluster, we calculated the mean thickness and SA, reducing the cortex to a total of 48 phenotypes. See Figure 2 for cluster visualization and labeling. See Extended Data Figures 2-1, 2-2, 2-3, 2-4 for correspondence between cortical vertices and SA or CT clusters. In this analysis, we preferred the clusters derived from our QTIM sample to those defined previously in a sample of middle-aged male veterans from the US (Chen et al., 2013). However, it is interesting to observe that the clusters are largely conserved across these two samples (Couvy-Duchesne, 2017).

10.1523/ENEURO.0153-17.2017.f2-1 Extended Data Figure 2-1 Description of vertices in each of left CT clusters. Download Figure 2-1, XLS file.

10.1523/ENEURO.0153-17.2017.f2-2 Extended Data Figure 2-2 Description of vertices in each of left SA clusters. Download Figure 2-2, XLS file.

**Figure 3.**
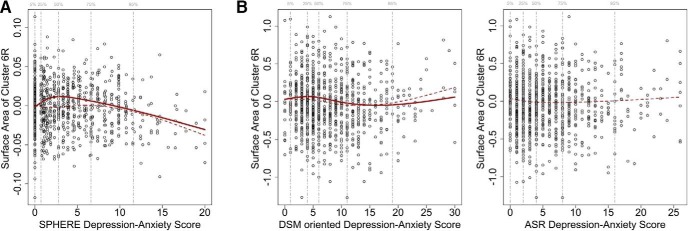
Nonlinear associations between anxiety-depression and SA of the genetic cluster 6R. The solid line represents the regression effect of the significant spline order. The dashed line combines the association for the linear, quadratic, and cubic splines and the anxiety-depression SPHERE score and the SA of the 6R cluster. The *y*-axis corresponds to SA after removing the effect of the intercept and all other covariates. The vertical dashed bars indicate the 5, 25, 50, 75, and 95% quantiles of the scores distributions. ***A***, Association between SA and cluster 6R SA and the SPHERE anxiety-depression score (QTIM sample). The reduction of SA is observed in participants with SPHERE score >2.9 (median score, see solid line: 3rd order natural spline). We interpret the increase of the (solid) regression line for SPHERE score below median as an artifact of plotting the significant spline order only. This effect is not observed when the three splines order are combined. ***B***, Association between SA for cluster 6R and the DSM-oriented (left panel) and ASR syndrome based (right panel) anxiety-depression scores. A consistent reduction of SA was observed in participants with ASR DSM-oriented scores between 4 and 19 (25-95% percentiles; see solid line: 1st order natural spline). No significant association was found with the ASR syndrome based scale.

10.1523/ENEURO.0153-17.2017.f2-3 Extended Data Figure 2-3 Description of vertices in each of right CT clusters. Download Figure 2-3, XLS file.

**Figure 4.**
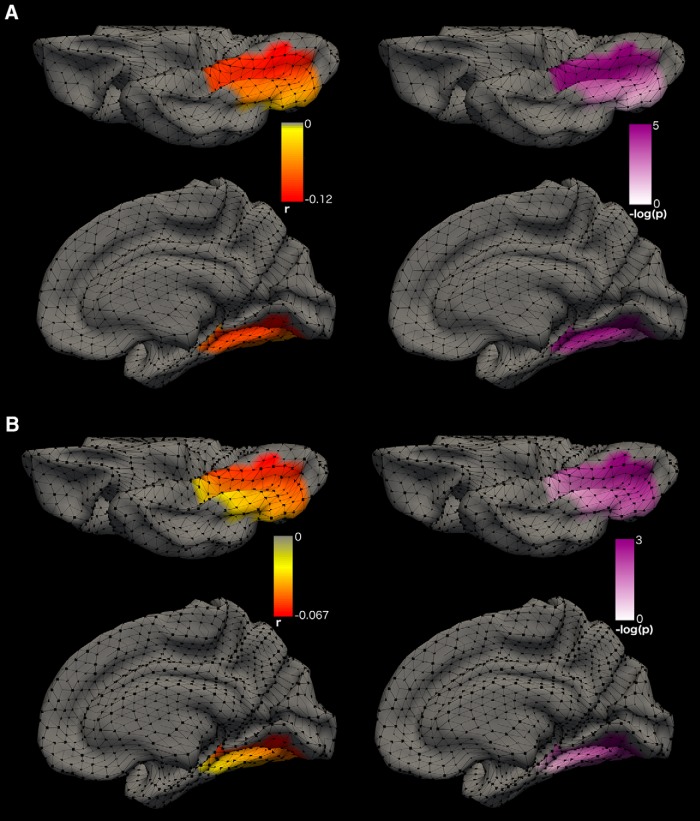
Vertex-wise phenotypic association of SA with anxiety-depression scores within cluster 6R. Bottom view (top) and medial view (bottom) of the right hemisphere. The left panels show the cubic effect sizes (correlations) for each vertex of the 6R cluster for SA (94 vertex). The right panels show the significance (*p* value) of the association in –log10 scale. Vertices represent the intersections of the triangular mesh reconstructed by FreeSurfer to model cortical surfaces. ***A***, Vertex-wise association between SA and anxiety-depression (SPHERE score) in the QTIM sample (*n* = 833). The significance threshold of 4.2E-4 is reached for *p* > 3.4 in the log scale. ***B***, Vertex-wise association between SA and anxiety-depression (DSM-oriented score) in the HCP sample (*n* = 890).

10.1523/ENEURO.0153-17.2017.f2-4 Extended Data Figure 2-4 Description of vertices in each of right SA clusters. Download Figure 2-4, XLS file.

#### Association between cortical measures and anxiety-depression

First, we tested the phenotypic association between the anxiety-depression SPHERE score and both SA and CT for each of the genetic clusters. To capture complex associations, we modeled linear, quadratic, and cubic relationships using natural splines, implemented in R (R Development Core Team, 2012). Natural splines are piecewise polynomials (of 3rd order here) that include constraints at the boundaries, forcing the tails of the association to be linear, thus preventing diverging “unnatural” solutions. All models included the three splines orders, required to make the results interpretable. This approach can detect both linear and U-shaped (quadratic) relationships, as well as changes limited to the extreme of the anxiety-depression continuum (cubic), or even a mixture of these. Compared to simple linear modeling, this should provide a better fit to the data and may detect associations otherwise overlooked. In addition, splines are more robust than polynomial regression as the splines orders are constrained to be orthogonal, thus removing issues arising from collinearity between the predictors. To limit the effect of outlier scores on the results, we winsorised four observations with SPHERE sum scores >20 (>4 SD away from the mean, in the heavy right tail).

To control for sample relatedness, we used a mixed model that integrates the variance-covariance of observations via a 834 × 834 kinship matrix (calculated with R package “kinship2”; Therneau and Sinnwell, 2015). To estimate the parameters of the models, we used the “hglm” package (Rönnegård et al., 2010) that relies on extended quasi-likelihood (Lee and Nelder, 1996; Lee and Lee, 2012; Rönnegård and Lee, 2013). The significance of the anxiety-depression score (and all fixed effects) was tested using Student’s *t* tests [test statistic: β/SD(β)]. We included as covariates the linear and nonlinear (quadratic and cubic) effects of age (at questionnaire and age at scan), sex, wave, acquisition direction (coronal or sagittal), as well as mean vertex-wise SA/CT to adjust for sex and age, which are in turn strongly associated with head/body size/cortical SA (sex, age) and thickness (age).

To avoid an overly stringent multiple testing correction, we estimated the effective number of independent brain phenotypes from the eigenvalues of the correlation matrix (Li and Ji, 2005). This method provides an accurate and fast alternative to permutation tests (Li and Ji, 2005). We estimated the effective number of genetic clusters to be 33 (out of a total of 48; Li and Ji, 2005), after regressing out the covariates (age, sex, acquisition, wave, mean vertex-wise SA/CT) that tend to inflate the coefficients of the correlation matrix. This translates into ∼99 independent tests (as we are testing three effects: linear, quadratic, and cubic), which yields a significance threshold of 5.2E-4 (Sidak, 1967) corresponding to a family wise error rate (FWER) less than or equal to 5%.

Then, we used bivariate (AE) twin models to estimate the genetic and environmental correlations between cluster measurements and anxiety-depression. Covariates used in the phenotypic association analysis were regressed from the brain measurement and the residuals were used in the twin analysis (for sample composition, see Table 1). In this article, we only used residuals in multivariate OpenMx models to reduce computational time and directly included the covariates in all other models. We tested the significance of the heritability estimates as well as the genetic and environmental correlations using a likelihood ratio test on nested models (1 degree of freedom tests). We corrected for the number o*f* tests performed. Twin analyses were performed in OpenMx (Boker et al., 2011). We also conducted a *post hoc* analysis to highlight which voxels from significantly associated genetic clusters were driving the phenotypic association with anxiety-depression.

#### Post hoc association analyses in the QTIM sample

In addition to the main analysis, we report the association of the SPHERE scores with the surface and thickness measurements derived from the Desikan anatomic atlas (Desikan et al., 2006), as well as a vertex-wise analysis. This facilitates comparison with previous studies and shows that the reported associations are not an artifact of our genetic parcellation. The same models and covariates were used as for the main analysis. We estimated the significance threshold to be 1.5E-4 (108 effective phenotypes) for the associations with the anatomic cortical regions. For the vertex-wise analysis, we used the brain-wide significance threshold (1.6E-5, based on 10,000 effective phenotypes) calculated by Medland et al. (2014). Both significance thresholds took into account that we tested three splines orders.

### Replication analysis

#### Participants

We used MRI and Achenbach ASR questionnaire (Achenbach, 2009) data for 890 participants (mean age 28 SD 3.7 range 22–37, 56.5% female) from the Human Connectome Project (HCP; Van Essen et al., 2012b, 2013) to replicate our results. In twin modeling, a maximum of 3 participants per family was included, resulting in 184 complete trios, 109 pairs, and 83 singletons (for detailed breakdown, see Table 1).

We utilized preprocessed T1-weighted structural scans from the HCP sample (Marcus et al., 2011). Minimal processing of the structural images by the HCP team consists of removing spatial artifacts and T1 alignment (Van Essen et al., 2012a; Glasser et al., 2013), using FSL (Jenkinson et al., 2002, 2012), and FreeSurfer (Fischl, 2012). We applied the same techniques used for QTIM data to produce the vertex-wise and genetic cluster measures from the preprocessed HCP surfaces.

We used the two ASR scores that were available: the anxiety-depression syndrome based scale (Achenbach, 2009) and a DSM-oriented scale that we constructed by summing the DSM-oriented scales for both anxiety and depression (Achenbach et al., 2003). The correlation between the DSM-oriented anxiety and depression scales was 0.66 (95%CI: 0.62–0.70) and mostly driven by common genetic sources of variance: rG = 0.87 (95%CI: 0.70–1.00), rE = 0.54 (95%CI: 0.42–0.64). These high phenotypic and genetic correlations suggest that combining the two DSM-oriented scales is a valid approach. Participants reported an average syndrome score of 5.7 (SD = 5.2, range 0–33) and an average DSM-oriented score of 8.0 (SD = 5.6, range 0–35). The phenotypic correlation between the DSM-oriented and syndrome anxiety-depression score was 0.90 (95%CI: 0.89–0.91), with comparable genetic and environmental correlations (rG = 0.91, 95%CI: 0.84–0.95, rE = 0.89, 95%CI: 0.85–0.92). Notably, the genetic correlation between the two scores was significantly different from 1 (*p* = 1.2E-4), suggesting that they may have some unique genetic sources of variance. All ASR scales have previously shown good test-retest reliability and internal consistency (Achenbach et al., 2005). In addition, the ASR syndrome based scale is heritable through adolescence (Nivard et al., 2015) and captures a stable construct across age and sex (Fonseca-Pedrero et al., 2012). DSM-oriented and syndrome based scales appear to comparably predict affective disorder diagnoses (Najman et al., 2008; Dingle et al., 2010, 2011).

#### Replication analysis

We considered for replication all significant associations between the anxiety-depression (SPHERE) score and genetic clusters identified in the exploratory analysis. As before, we tested for linear, quadratic, and cubic association with the brain phenotypes. We also corrected for sex, acquisition variables, total SA or CT, linear and nonlinear effects of age as well as familial relatedness using a kinship matrix (Therneau and Sinnwell, 2015). We controlled for multiple testing in the replication analysis by estimating the effective number of independent phenotypes carried in the replication step (Li and Ji, 2005). We further corrected for testing linear, quadratic, and cubic relationships and for considering the two ASR anxiety-depression scores. ASR scores >4 SD from the mean were winsorised to limit the influence of outliers (heavy right tail) on the results.

We then explored the voxel-wise associations within replicating clusters and further decomposed the associations into their genetic and environmental components using bivariate AE twin models in OpenMx (Boker et al., 2011). As before, we regressed out the covariates before twin modeling.

#### Code accessibility

The code/software described in the article is freely available online at [https://github.com/baptisteCD/Lingual-Gyrus-surface-area-is-associated-with-anxiety-depression-severity-in-young-adults-a-genetic]. The code is also accessible as Extended Data Figure 1.

10.1523/ENEURO.0153-17.2017.ed1 Extended Data 1 R code used for all the analyses. Download Extended Data, ZIP file.

## Results

### Exploratory analysis

Across the 48 brain clusters only one (cluster 6R for SA) comprising the lower part of the occipital cortex was associated with the anxiety-depression score from the SPHERE, after correcting for multiple tests (3rd order spline association: β = −0.037, SD = 8.6E-3, rP = −0.10, *p* = 2.4E-05; Table 2). SA in this cluster appeared to peak for participants with a median SPHERE score (score = 2.8) and to be substantially decreased (up to 1 SD lower) in those with a high-anxiety depression score (Fig. 3*A*). However, we should be cautious interpreting the increasing SA for participants with low SPHERE scores, as it may be an artifact of plotting only the significant spline order. Indeed, this increase is not observed when plotting the effect of all splines order (Fig. 3*A*, dotted line). We are confident the model is not over fitted as the three splines order only accounted for a 1.6% of the score variance and we did not observe large standard errors for the estimates, which often indicate colinearity.

**Table 2.** Summary of discovery (QTIM sample) and replication (HCP sample) analysis

	**First order spline (linear)**		**Second order spline (quadratic)**		**Third order spline(cubic)**	
	β (se) r	*p* value	β (se) r	*p* value	β (se) r	*p* value
**QTIM**						
**SPHERE scale**	0.010 (6.0E-3) *r* = 0.030	0.094	−0.021 (6.5E-3) *r* = −0.069	1.6E-3	**−0.037 (8.6E-3)** ***r* = −0.10**	**2.4E-5**
**HCP**						
**DSM-oriented scale**	**−0.18 (0.060)** ***r* = −0.040**	**3.2E-3**	0.015 (0.12) *r* = 0.0022	0.89	0.15 (0.120) *r* = 0.022	0.13
**ASR syndrome based scale**	−0.032 (0.076) *r* = −0.0064	0.68	−0.044 (0.10) *r* = −0.0088	0.67	0.079 (0.15) *r* = 0.012	0.61

Significant associations (after multiple testing correction) are reported in bold.

Fitting AE models, heritability was estimated to be 0.55 (95%CI: 0.32–0.73) for the 6R cluster for SA and 0.28 (95%CI: 0.14–0.41) for anxiety-depression (SPHERE), but neither the genetic nor environmental correlations between these traits reached significance (cubic correlations: rG = −0.068, *p* = 0.35 and rE = −0.084, *p* = 0.11; linear correlations: rG = −0.20, *p* = 0.35 and rE = 0.0091, *p* = 0.91). In comparison, heritability of the other SA clusters ranged from 0.39 to 0.71 (0.32 to 0.63 for the CT clusters).

The 6R cluster for SA consists of a total of 94 vertices and is located in the occipital cortex. It comprises most of the fusiform gyrus (56.6% or 43 vertices) and the parahippocampal gyrus (56.5% or 13 vertices) as well as the lower part of the lingual gyrus (33.8% of the gyrus or 22 vertices) and the medial part of the lateral occipital cortex (12.6% or 12 vertices). To identify if one or more regions of the 6R cluster were driving the cubic association between SA and the anxiety-depression SPHERE score we restricted our analysis to the 94 voxels in the 6R cluster. Using a significance threshold that accounts for the extra number o*f* tests performed (*p* < 4.2E.4), 61 voxels were found to be driving the nonlinear association between SA of the cluster 6R and the anxiety-depression SPHERE score (*p* range: 5.9E-06 to 3.8E-04, correlation range: −0.13 to −0.091; Fig. 4*A*). When decomposing the phenotypic association using an AE model, none of the genetic or environmental correlations was significantly different from zero.

### Replication analysis

We found a significant (1st order spline) association between the DSM-oriented score for anxiety-depression and SA of the 6R cluster (Table 2), using a significance threshold of 8.5E-3, which corresponds to a FWER < 5% considering 6 independent tests (3 tests of association for two anxiety-depression scores). This association supports our finding in the QTIM data set, of a reduction of SA (*r* = −0.040, *p* = 3.2E-3) in participants with moderate anxiety-depression score (25th–95th percentile; Fig. 3*B*), which we reported above.

In the HCP dataset, SA for the 6R cluster was moderately heritable (0.63; 95%CI: 0.52–0.72), after regressing mean SA and the other covariates, as well as the anxiety-depression scores: 0.36 for the DSM oriented ASR (95%CI: 0.20–0.51) and 0.41 for the syndrome based ASR (95%CI: 0.24–0.56). When breaking down the phenotypic association between DSM-oriented scale and 6R SA, neither genetic nor environmental components reached significance (linear correlations: rG = −0.090, *p* = 0.37 and rE = −0.032, *p* = 0.59). Likewise, when using the ASR syndrome based scale (rG = −0.078, *p* = 0.56 and rE = −0.059, *p* = 0.49). Heritability of other SA clusters ranged from 0.46 to 0.82 in the HCP (0.42 to 0.72 for CT clusters).

We then tested the association between each voxel of the cluster with the DSM-oriented scale. We estimated the number of effective independent voxels to be seven (Li and Ji, 2005) after regressing out the covariates’ effect on the vertex-wise measurements. Thus, we used a significance threshold of 3.6E-3, to account for 13 independent tests (six previously and seven for the voxel-wise testing). Nineteen (out of 94) voxels located in the medial posterior part of the cluster survived multiple testing correction (-log(*p*)>2.4, r in −0.040 −0.068; Fig. 4*B*). Thirteen of these were located in the lingual gyrus and were also associated with the anxiety-depression scores in QTIM. A further three neighboring vertices from the fusiform gyrus were also consistently associated to anxiety-depression across the two samples (Table 3). As previously, vertex-wise genetic and environmental correlations did not reach significance.

**Table 3.** Summary of *post hoc* vertex-wise association testing for SA of the 6R cluster

	***N* total vertices in each gyrus**	***N* vertices for cluster 6R**		***N* vertices associated in vertex-wise analysis- QTIM**	***N* vertices associated in vertex-wise replication analysis – HCP**
		*N*	MNI coordinates		
**Parahippocampal**	23	13	*x* in 14 33 *y* in −64 −50 *z* in −19 −6	13	0
**Lingual**	65	22	*x* in 7 32 *y* in −107 −68 *z* in −4 6	22	13#
**Fusiform**	76	43	*x* in 24 43 *y* in −102 −58 *z* in −19 3	22	3*
**Lateral occipital**	95	12	*x* in 21 45 *y* in −109 −91 *z* in −8 0	0	2

# All vertices associated in QTIM. Vertices correspond to numbers 144, 570, 573, 2220, 2244, 2252, 2253, 2258, 2262, 2264, 2265, 2266, and 2269 of the FreeSurfer fsaverage4 parcellation (MNI coordinates: *x* in 7 20, *y* in −107 −82, and *z* in −3 6).

* All vertices associated in QTIM. Vertices correspond to number 2263, 2267, and 2270 of the FreeSurfer fsaverage4 parcellation (MNI coordinates: *x* in 24 27, *y* in −98 −92, and *z* in 0 3).

### *Post hoc* association analyses

The association analysis between the SPHERE scores and anatomic regions from the Desikan atlas returned one significant association with SA of the right lingual gyrus (*p* = 9.5E-5). As per the main QTIM analysis, the association was negative and with the 3rd order spline (β = −330.7, SD = 83.9, *r* = −0.13). No significant association was found at a vertex-level for SA or CT.

## Discussion

Here, we used twin modeling to derive a genetic parcellation of two characteristics of the cortical ribbon (i.e., SA and thickness), reducing each hemisphere to 12 measurements. The SA of one region (cluster 6R) comprising part of the lingual, fusiform, parahippocampal and lateral occipital gyri showed a significant association with the anxiety-depression score from the SPHERE in the QTIM sample. We partially replicated this association in an independent sample of similar age, using data from the HCP, in which we found an association between the DSM-oriented score and SA in the 6R cluster. Both associations suggested lower SA in participants with anxiety-depression scores above average (50–95% quantiles). A *post hoc* vertex-wise analysis suggested that the lingual vertices (and to a lesser extent fusiform) might drive the association. We cannot conclude whether the observed association is specific to the right hemisphere due to differences in the cortical parcellation between hemispheres (Fig. 2; in the left hemisphere the occipital cortex is composed of a unique cluster).

Interestingly, a meta-analysis (472 cases, 680 controls, from 12 published studies, mean age 43, partially medicated) identified significant reductions of GM density (Ashburner and Friston, 2000; often considered as a proxy for volume) in right lingual, fusiform and parahipocampal gyri (Arnone et al., 2016). The authors found little evidence for publication bias and the voxel-based morphometry approach cannot be confounded by head/body size. This echoes results from the meta-analysis by the ENIGMA-MDD group, which reported a significant reduction of right lingual gyrus SA in adolescent depression (237 cases, 294 controls; Schmaal et al., 2017b; nonsignificant association for fusiform or parahippocampal). Although the meta-analysis by ENIGMA did not correct for total SA and included medicated cases, it is also not totally independent from the present study as it included QTIM participants (26 cases, 140 controls). Even so, in other work, right lingual GM density was identified as a predictor of anti-depressant response, with higher density predicting better response to first treatment (Jung et al., 2014). The nonrespondent group showed lower performance in three aspects of the Stroop test (neutral word, color word and error control), and a *post hoc* analysis in cases showed right lingual density to be associated with better error control (Stroop), and nonverbal memory (Rey-Kim memory test). This is coherent with neurologic case reports that highlight the crucial role of the lingual gyrus in visual memory (Bogousslavsky et al., 1987). Furthermore, impaired visual memory has been associated with 1st episode MDD (Lee et al., 2012) and with adolescent MDD (Baune et al., 2014); lower Stroop accuracy has also been reported in adult MDD (Snyder, 2013), while more research is needed in pediatric depression (Vilgis et al., 2015). Finally, reduced right lingual volume was also reported in adult MDD cases (partially medicated; Lee et al., 2011), following traumatic brain injury (Maller et al., 2014).

The reported right lingual gyrus volume (or density) reduction in depression may be driven by reduced SA, at least in early adulthood, which may be associated with visual memory and attention deficits in depression. In addition, the right lingual SA may be associated with anti-depressant response and cognitive functions in MDD patients (Jung et al., 2014), which might explain why the findings are not consistent across samples that may differ in term of medication status, response to treatment or cognitive abilities. For example, screening in QTIM may exclude individuals with extreme levels of anxiety-depression, and we may be only observing part of the association.

Reduction of right fusiform gyrus volume has also been reported in off-medication adult patients (Lener et al., 2016), but several large studies of older (medicated) participants attributed this reduction to reduced CT (Canu et al., 2015; Schmaal et al., 2017b). In addition, CT of the right fusiform in MDD cases with comorbid generalised anxiety was even more reduced (Canu et al., 2015). The right fusiform gyrus has a central role in face perception (Haxby et al., 2000), in neurologic case reports of prosopagnosis (face blindness; Whiteley and Warrington, 1977; Damasio et al., 1990; De Renzi et al., 1994), behavioral studies (Rhodes, 1993) and imaging studies (McCarthy et al., 1997; Haxby et al., 2000). Lower right fusiform gray matter density was observed in developmental prosopagnosis (Garrido et al., 2009) and in congenital prosopagnosia (Behrmann et al., 2007). In addition, both studies reported an association between right fusiform volume and performance in face identification (Behrmann et al., 2007; Garrido et al., 2009). Reviews on adult depression suggest a generally reduced accuracy of face expression evaluation, coupled to increased attention and response bias in expression evaluation toward sadness (Bourke et al., 2010).

In adolescent depression, more research is needed to confirm the mixed evidence of impairment in face (and face expression) processing (Vilgis et al., 2015). Lower fusiform gyrus SA in young adults might relate to the poorer face recognition and processing of face expressions reported in adult depression but we did not have the cognition data to test such hypothesis. However, our results only weakly point to the fusiform gyrus and do not align with prior studies that only reported a reduced fusiform thickness associated with MDD (Canu et al., 2015; Schmaal et al., 2017b). Overall, we cannot rule out that medication or sample age might explain some of these differences, as well as the hypothesis of delayed cortical development in depressed participants, implying a normalization of cortical surface in mid-adulthood that leaves a more permanent decrease in thickness (Schmaal et al., 2017b).

In line with prior publications (Panizzon et al., 2009; Chen et al., 2013) that reported significant heritability of CT and surface, we confirmed that the SA of the 6R cluster is significantly heritable (h2_QTIM_ = 0.55, h2_HCP_ = 0.63), even after correcting for total SA, suggesting that this region is influenced by specific genetic sources of variance, that do not contribute to global SA. In addition, all anxiety-depression scores were comparably heritable (h2_SPHERE_ = 0.28, h2_DSM-ASR_ = 0.36, h2_ASR_ = 0.41), in line with the known heritability of depression from twin and family studies (Sullivan et al., 2000; Kendler et al., 2006; Polderman et al., 2015). In the bivariate analysis, because of the modest phenotypic association, we had limited statistical power to determine whether common genetic or environmental factors drove the observed phenotypic association. Indeed, a *post hoc* power analysis indicates that we had, at best, 10% power to detect the observed genetic correlation in QTIM and HCP (taking into account observed heritability and effect sizes, assuming an AE model and a risk α = 5%). A combined analysis of the two samples would not confer a statistical power >22%. The limited power may explain the instability of the correlations estimated in both samples, even if differences in anxiety-depression questionnaire, sample composition or recruitment could also be at play.

The reported heritabilities of the scores are consistent with the diathesis-stress model of anxiety-depression, in that genetic liability and environmental factors both contribute to the risk of depression (Rosenthal, 1963), sometimes in a multiplicative manner (Peyrot et al., 2014; Musliner et al., 2015; Mullins et al., 2016; Colodro-Conde et al., 2017). More generally, the identification of brain endophenotypes should lead to refine this model by providing insight into the brain networks and behavioral mechanisms that are influenced by the genetic and environmental risk factors. We still lack evidence to provide a credible interpretation for the association reported here, which may require reconciling evidence produced from complementary types of imaging. For example, reduced glucose metabolism in the right lingual gyrus has been linked to sleep anomalies in depression (Germain et al., 2004; Fitzgerald et al., 2008) but more work is need to investigate whether structural cortical markers may tag the same brain pathways and contribute to the same symptoms.

Our study has several limitations. Firstly, we did not perform a strict replication as QTIM and HCP differed in the anxiety-depression scores used, time difference between scoring and scanning, and shape of the relationship (3rd vs 1st order splines). Thus, differences in studies design (different timeframes, medication, population genetics and environment, ascertainment bias) might be partially responsible for the different effect size observed in QTIM and HCP. In addition, the SPHERE and ASR both aim to measure anxiety-depression but they are composed of different questions and may be sensitive to different ranges of symptoms or different stages of the disorder (e.g., subclinical for the SPHERE and clinical for the DSM based ASR). Furthermore, the association between 6R SA and anxiety-depression scores in the HCP was only observed using the DSM-oriented score, which we attribute to differences in scores distribution making it harder to detect nonlinear associations. Indeed, despite high (phenotypic and genetic) correlations between the scores (r = 0.90, rG = 0.91), the correlation between spline orders was lower (r = 0.79 between 1st order splines of the two anxiety-depression scores, 0.56 between 2nd order and 0.73 between 3rd order). This may reflect differences of scale and distribution. Differences in genetic sources of variances (genetic correlation different from 1) are unlikely to completely explain the differences in results, as the correlation remained high. Nonetheless the different results between QTIM and HCP as well as the inconsistent associations in the HCP sample using different scores remain important limitations of this study. These limitations call for further investigation of the relationship between lingual gyrus and anxiety-depression.

The small effect size of the association in QTIM and HCP can also be seen as a limitation, as lingual gyrus SA does not explain much of the anxiety-depression variability and would have a very limited predictive power. However, our result is in line with the effect sizes observed in larger case-control studies that suggest that no single brain marker of depression explains much of the disorder (Schmaal et al., 2017b). A parallel to this is the small SNP effects observed/found in GWAS of complex traits (Visscher et al., 2017). Thus, despite the small association between cortical regions and the trait/disease, their impact on brain networks and functions may be much larger. That is, a small association does not necessarily preclude a biological or cognitive relevance.

Further limitations include our clustering approach, which may make it harder to detect localised structural changes associated with anxiety-depression, as the thickness and surface are averaged over large cortical regions. In addition, we did not investigate sex specific changes that may reflect depression subtypes with different etiology (Kendler et al., 2001; Kendler and Gardner, 2014). As per the hypothesis of age specific markers reported by the ENIGMA-MDD (Schmaal et al., 2017b), age was not significant in our analysis, which prevented us from testing for an age interaction with anxiety-depression scores. However larger samples would be required to overcome the multiple testing correction burden from a vertex-wise analysis or the loss of power resulting from study stratification. Finally, the interpretation of our results is limited by the relative lack of robust research on cognitive and imaging aspects of pediatric depression and of normal neurodevelopment. The Research Domain Criteria (RDoC) may help in making connections between cognition, imaging, genetics, and psychiatric illnesses.

In summary, we proposed a candidate endophenotype for depression, which is heritable, and shows a replicable phenotypic association with anxiety-depression scores. The vertex-wise *post hoc* analysis suggested that a reduction of SA in the ventral part of the lingual gyrus could drive the observed association. Longitudinal studies beginning in adolescence are emerging (Luby et al., 2016; Schmaal et al., 2017a) and will help clarify the temporal and causal relationships between brain development, cognition and mental health.

## Synthesis

Reviewing Editor: Menno Witter, Norwegian University of Science and Technology Decisions are customarily a result of the Reviewing Editor and the peer reviewers coming together and discussing their recommendations until a consensus is reached. When revisions are invited, a fact-based synthesis statement explaining their decision and outlining what is needed to prepare a revision will be listed below. The following reviewer(s) agreed to reveal their identity: Jeremy Elman.

The authors have adequately responded to the two reviews of the previous version of the paper and this version has been favourably evaluated by one reviewer. However, we feel that the paper would benefit from some additional work, as indicated below.

The current study attempts to find endophenotypes of anxiety-depression symptoms. The authors first generate genetic parcellations of cortical thickness and surface area in a large twin study. Next, they test for relationships with an anxiety-depression scale and further test for genetic correlations between these measures. A similar analysis is conducted within a replication study. They do find a non-linear relationship between the anxiety-depression scale and surface area in a parcellation largely covering the lingual gyrus. In the replication sample, there is a linear relationship between the anxiety-depression scale and surface area in the same genetic parcellation.

The methodology described in this paper is quite strong, and the authors have rigorously controlled for false positives. While the use of a replication sample is laudable, the inconsistent patterns indicate that the effect is either not reliable, or not robust. Unfortunately, these results diminish the compelling nature of the study. Concerns are outlined below:

1. The most obvious pattern apparent in Figure 3 is that the relationship between depression-anxiety scores and surface area is reversed in participants above the mean (the linear and cubic splines coefficients even have different signs across studies). If the goal of this study is to find an endophenotype of psychiatric disorder, these individuals with more severe symptoms would seem to be the most relevant subset, and thus the inconsistent relationship in this range is particularly important. The authors have discussed some potential explanations for this, such as differences in the samples and methodology. This presents to possibilities: 1) If these samples and measures are equivalent, the effect is not very reliable; or 2) The effect is not robust to sample and methodological differences that are likely to occur across studies. In either event, it calls into question the usefulness of this measure as an endophenotype more broadly.

2. Related to the above point, are the measures of depression-anxiety used in the two studies though to be comparable? Are they more or less sensitive to different ranges of symptoms?

3. The authors make an argument for why there is reason to examine a cubic spline, but given the lack of replication, it does raise the likelihood that the model was simply overfit in the first sample.

4. I'm not sure the argument that differences in effect could arise from differences in medication status. I believe the study they cite suggests greater lingual gyrus volume can predict better medication response, not that medication causes increased lingual gyrus volume.

5. Why did the authors choose to generate a set of genetic parcellations rather than use the set reported in the Chen et al. (2013) study? It might be useful to describe how closely the current parcellation resembles this previous set.

6. The vertex-wise analysis within clusters doesn't seem to add much information. While it may give a sense of whether certain voxels are driving the result, but given the size of the parcellations, is much regional heterogeneity to be expected? Of course, there's nothing wrong with this analysis, but a whole brain vertex-wise analyses (even one showing uncorrected effect maps) might be more informative from a descriptive standpoint.
